# Space use of Pacific harbor seals (Phoca vitulina richardii) from two haulout locations along the Oregon coast

**DOI:** 10.1371/journal.pone.0219484

**Published:** 2019-07-31

**Authors:** Sheanna Steingass, Markus Horning, Amanda M. Bishop

**Affiliations:** 1 Oregon State University Department of Fisheries and Wildlife, Oregon Department of Fish and Wildlife, Corvallis, Oregon, United States of America; 2 Alaska SeaLife Center, Seward, Alaska, United States of America; 3 Oregon State University Department of Fisheries and Wildlife, Newport, Oregon, United States of America; Pacific Northwest National Laboratory, UNITED STATES

## Abstract

**Background:**

There are approximately 10,000–12,000 Pacific harbor seals (*Phoca vitulina richardii*) inhabiting the Oregon coast, and unlike other species of pinnipeds in this region, are reliably present year-round. Despite this, and drastic rebounds in population since the enactment of the Marine Mammal Protection Act, limited data is available for the present period regarding their space use at sea, and within estuarine, riverine, or bay areas within the state.

**Objective:**

To examine site-based differences in space use for 24 adult Pacific harbor seals captured and outfitted with satellite transmitters at two predominant haulout sites on the Oregon Coast, USA.

**Design:**

We captured 24 adult harbor seals from two haulout sites on the Central Oregon coast between September 2014–16 and fitted them with external Wildlife Computers SPOT5 satellite transmitters to track movement. Using state-space modeled locations derived from satellite telemetry data, we evaluated spatial behavior of these animals using a correlated random walk model via R package crawl. Kernel density estimation was subsequently used to calculate home range and core area for each animal. Percent use of open ocean habitat versus use of estuaries, rivers and bays was quantified, as was an initial examination of presence within five newly-established marine reserves in Oregon. Examination of haulout site-related differences in spatial behavior were examined for seals captured in Netarts and Alsea Bays, Oregon and haul out behavior related to time of day, season, and tidal level was also investigated.

**Results:**

The average individual home range for seals was 364.47 ± 382.87 km^2^ with seals captured in Alsea bay demonstrating a significantly higher home range area than those captured in Netarts Bay. Alsea bay seals also tended to range farther from shore than Netarts Bay animals. The average calculated core area for seals encompassed on average 29.41 ± 29.23 km^2^ per animal, however the home range of one animal was so small, core area could not be calculated. Use of marine reserves was limited for animals in this study, representing less than 2% of locations with a majority occurring in Cape Perpetua Marine Reserve and North Marine Protected Area. Seals were more likely to haul out during low tides and periods of low light (dusk, night and dawn), and hauling out behavior increased in winter months.

**Significance:**

These findings demonstrate the first major documentation of space use of harbor seals in the state for nearly three decades, and lends itself to future comparison and formation of mechanistically-based hypotheses for behavior of a common marine mammal in the highly productive northern California Current System.

## Introduction

Pacific harbor seals are a common generalist predator and one of the most populous pinniped species in Oregon. Unlike other pinnipeds (specifically otariids) in this area, harbor seals do not undertake extensive migrations and are reliably present in the region year-round [1]. Prior to the Marine Mammal Protection Act in 1973 harbor seals were hunted to near-extinction within the state. Population estimates taken in 1967 and 1968 revealed approximately 500 individuals along the entirety of the Oregon coast [2,3]. This was largely due to state-issued bounties placed upon harbor seals, which encouraged harvesting of animals that were seen as pests to fisheries. The $5–25 bounty payment resulted in hundreds of animals being harvested annually in the 1930s and 40s, until that number dropped drastically in the 1960’s. Abundance estimates from 2014 suggest that populations have rebounded to near carrying capacity (K), numbering approximately 11,565 (11,000–12,000) harbor seals in Oregon [4], and approximately 13,692 are present within Washington State [1]. Pacific harbor seal haulouts can be found throughout most of the Oregon coast at 91 surveyed locations [2,5]. These areas include natural bays, spits, mud flats and rocky intertidal zones, and in some cases anthropogenic structures. Harbor seals tend to forage on the nearshore continental shelf (<200m depth) on a wide variety of prey. They exhibit strong inter-individual variability in dietary composition and spatial behaviors [6–8], but are generally considered to display a high degree of site fidelity, returning to the same haulout(s) between foraging trips [9–11].

The at-sea habitat use of Pacific harbor seals has not been examined thoroughly within the latitudinal bounds of the 363 mile Oregon coast, which represents a significant section of the Northern California Current Large Marine Ecosystem of the Eastern Pacific. While the population distribution [12,13], foraging ecology [14–16] and dietary composition [17,18] of harbor seals have been well-studied in Washington, California [19–22] and Alaska [23–28] the only current existing data for harbor seals in Oregon relates to hard-parts based dietary studies [29–35]. One satellite telemetry study is available [36], but was restricted to tagging and movements within and around the Columbia River basin. Therefore, a clear need exists for baseline data that can be utilized for future comparison and for the development of hypotheses regarding the ecological role and behavior of these animals in their marine environment. Questions also remain as to the degree of site fidelity, behavioral differentiation, and areas of importance for harbor seals along the Oregon coast.

The geomorphology of the Oregon coast lends itself to dynamic biological processes, including strong annual upwelling events [37,38], a continental shelf with a high degree of variability in longitudinal extent from the shoreline [39], and strong seasonal and inter-annual productivity [37]. Oregon also has five newly-established marine reserves as of 2012, created in conjunction with eight marine protected areas, one seabird protection area, and nine associated comparison areas. Basic understanding of the space use of animals in this region is a key consideration for understanding comprehensive ecological function of management and conservation areas.

Here, we utilize satellite telemetry data gathered from 24 adult Pacific harbor seals captured at two sites on the Oregon coast from September 2014 to May 2016 to describe the general spatial habitat use by Pacific harbor seals in this understudied portion of their range. To do this we 1) define and describe home range and core areas for Pacific harbor seals from these sites, 2) quantify hauling out behavior and use of the open ocean versus riverine, bay and estuarine environments by study animals, and 3) examine intersite differences is spatial use from animals captured at two prominent but geologically-different haulout locations.

## Materials and methods

### Capture and tagging

Adult Pacific harbor seals (23 males, one female) were captured in Alsea (44.4279°N, 124.0679°W, n = 16) and Netarts (45.4028°N, 123.9484°W, n = 8) Bays on the coast of Oregon, in September 2014, 2015 and April 2015 (Table 1). We opted to include the adult female in our data analysis as her total deployment days, home range area, and trip metrics fell well within the range of other sampled animals.

**Table 1:** Individual animal ID, capture location and season, total period of transmission, and resultant locations after applying a regularized state space model.

PTT (ID#)	Body Mass (kg)	Capture Location	Capture Season	Capture Year	Deployment Period (Days)	SSM Locations
61694	84	Alsea	Fall	2014	111	1927
61695	82	Alsea	Fall	2014	212	2848
61764	66	Alsea	Fall	2014	172	2661
61765	77	Alsea	Fall	2014	231	3288
61766	55	Alsea	Fall	2014	264	4114
61767	57	Alsea	Fall	2014	250	3759
61768	90	Netarts	Fall	2014	112	1956
61769	70	Netarts	Fall	2014	159	2332
61774	87	Netarts	Fall	2014	112	1967
61775	82	Netarts	Fall	2014	324	4208
61776	68	Netarts	Fall	2014	51	1227
44611	108	Alsea	Spring	2015	56	1330
44613	71	Alsea	Spring	2015	143	3425
44614	69	Alsea	Spring	2015	116	2786
44615	53	Alsea	Spring	2015	44	1039
61698	116	Alsea	Spring	2015	56	1339
61754	98	Alsea	Spring	2015	112	2679
61770	86	Alsea	Spring	2015	96	2292
61771	105	Alsea	Spring	2015	42	991
61772	104	Netarts	Spring	2015	66	1587
61773	112	Netarts	Spring	2015	65	1543
61777	98	Netarts	Spring	2015	20	474
61778	NA	Alsea	Fall	2015	89	2133
61779	NA	Alsea	Fall	2015	222	5315

Adult seals >50 kg were targeted as ideal study animals to in an effort to reduce ontogenetic-related variability in behavior. Seals were captured with skiff-based purse seines or via hoop net beach rush methods by teams of 5–13 biologists including veterinary staff [40]. Animals were then individually weighed in hoop nets suspended from a tripod with a 0–200 x 1.0 kg hanging scale, then restrained on a custom-constructed fiberglass board during tag attachment. An external Wildlife Computers Satellite SPOT5 tag was attached to the post-cranial dorsal pelage with Devcon 5-minute epoxy or Loctite 422 adhesive [41]. SPOT5 tags are Argos transmitters that allow the satellite service provider (CLS America Inc.) to estimate transmitted locations via the Doppler shift. Location data are provided with associated classes based on estimated accuracy that range from 150m (LC3) to >1.5km (LCB) or unspecified error (LC Z) [42].

### Tag programming

For the first two tag deployment events (September 2014 and May 2015), tags were duty cycled with an on-off monthly cycle to provide a longer battery life, and thus promote data overlap between tag deployments (Table 2). Tags were sequentially set to ‘ON’ during April-May and September-October to coincide with the spring and fall transitions of wind-driven upwelling and downwelling [43]. Tags deployed in September 2015 were programmed to transmit continuously. Periods without transmission were not interpolated, but rather each active transmission cycle was treated as a separate set of points for analysis to minimize generating pseudolocations where there were no data. Tags were programmed to begin transmission to the Argos satellite array upon sensing immersion in salt water. To further extend battery life and to avoid over-representation of haulout periods, they were also programmed to reduce transmission rate after 10 consecutive ‘dry’ transmissions, and to temporarily halt transmitting after two hours of being dry until the next immersion in saltwater.

**Table 2:** Duty cycle programming for SPOT5 satellite tags.

	Jan	Feb	Mar	Apr	May	Jun	Jul	Aug	Sep	Oct	Nov	Dec
F14, S15	OFF	ON	OFF	ON	ON	OFF	ON	OFF	ON	ON	OFF	ON
F15	ON	ON	ON	ON	ON	ON	ON	ON	ON	ON	ON	ON

F14 = Fall Tagging 2014 (Sept. 9–10), S15 = Spring Tagging 2015 (April 7–8), F15 = Fall Tagging 2015 (Sept. 28).

### Parameter estimation

After tags permanently ceased transmitting, all data collected by the Argos satellite service provider CLS America, Inc. were downloaded via the Wildlife Computers Data Portal (Wildlife Computers). Locations categorized LC-Z were discarded due to lack of a measurable error estimate, and a burnout period of two locations at the beginning and end of deployment were used. Maximum swim speed for Pacific harbor seals is not widely-documented; therefore, a coarse speed-distance-angle filter (R package: argosfilter) was applied to the data in order to remove locations which required a biologically-unlikely travel speed of >8m/s and filtering angle of (-1°) and distance limits of (2500,5000m) [44]. The remaining raw datapoints were used to create a single-state movement model (State Space Model—SSM) for each animal using the R package *crawl* [45,46]. Each track segment was iterated 500 times each to create an average generated point for each location. *Crawl* uses the observed locations to interpolate a movement path by estimating locations at specified time intervals.

To determine the best interval for estimation we calculated the average time between consecutive ‘wet’ locations during active duty cycling periods, as determined by a majority of locations being ‘wet’ within one subsequent Wildlife Computers’ 20-minute timeline bin. The resultant mean inter-location time period was 1.52±9.83 hours. Based on this information, a temporal resolution of one hour was used to estimate a regularized movement path for each active duty cycle. Gaps in duty cycling were not interpolated and for that reason, behavioral data are not available and interpreted for those specific months. SSM outputs were further adjusted for unlikely presence on land by utilizing the ‘fixpath’ function in the R package *crawl* [45]. Fixpath utilizes a transition matrix to correct animal locations to obstruct travel through identified restriction zones, in this case over land identified as a coastline raster layer. Once the state-space modeled and the filtered datasets were generated, the resultant SSM-derived locations were used for all further analyses.

### Utilization distribution analysis

Kernel analysis was used to estimate individual utilization distributions (UD) for each animal at the 95% isopleth and every tenth isopleth from 10–90% using the Geospatial Modeling Environment (Spatial Ecology LLC) [47]. Home range was quantified as the 95% utilization isopleth [48]. Core area was calculated using methods from Vander Wal and Rodgers (2012) in order to estimate a biologically-relevant threshold, rather than using the standard 50% utilization distribution [49]. To generate this threshold, we utilized an exponential equation to identify the isopleth at which home range area began to increase at a greater rate than probability of use [50]. Core area was rounded down to encompass the nearest calculated 10% isopleth. To determine whether individual home range size (95% UD) was significantly positively correlated to the number of locations collected and therefore deployment duration, a linear model of log(95% UD (km^2^)) as a function of these two predictors was created and estimated using R’s linear model (lm) function. The 95% UD area (response variable) was log-transformed after examination of non-normality by the Shapiro-Wilkes test.

### Hauling out and use of riverine and estuarine locations

The boundaries of estuaries, bays or rivers were defined as the area within the two farthest-seaward points of land for each bay or river, including jetties. Utilization of bay, riverine and estuarine waters was categorized into the binary variable of presence or absence and points within each area (‘present’) were further divided into wet or dry status, indicating time in water or hauled out based on the tag’s wet/dry timeline. Excluded from this analysis were the final two trips from seal #61779, which spent the winter within the Columbia River, resulting in a data gap due to lack of salt water to activate the tag. Area use for bays and rivers was calculated using the spatial join feature in ArcGIS, and calculated as percentage of total points inside a feature. In order to determine characteristics of in-water trips versus haulout periods, the wet-dry timeline was utilized to identify unique ‘trips’. Points were treated as single point locations rather than tracks, but were organized into individual trips based on tag transmission cycles using two criteria. Foraging trips after leaving the haulout were identified as the first ‘wet’ location within a transmission period, through the last consecutive ‘wet’ location before a ‘dry’ status ended the trip. Additionally, time in bays, rivers, or estuaries was characterized by time wet vs. dry (hauling out) within these habitats.

The dynamic oceanographic variable of hourly tidal height (m) was extracted for all data points from the NOAA Tides and Currents Data Server for the South Beach, Oregon oceanographic mooring (Station #9435380, 45°N, 125°W) and matched to the nearest hour of each SSM-generated location. Daily upwelling index was obtained from the Pacific Fisheries Environmental Laboratory (PFEL) live access server for the South Beach, Oregon oceanographic mooring (Station #9435380, 45°N, 125°W) and matched to date for each point (https://www.pfeg.noaa.gov/products/PFEL/modeled/indices/upwelling/upwelling.html). PFEL generates coastal upwelling indices (CUI) using the magnitude of Ekman Transport (wind stress divided by the Coriolis parameter). Stronger equatorward (northly) wind stress, which drives upwelling, is denoted by higher positive values of the CUI. Stronger poleward (southerly) wind stress, which drives downwelling, is denoted by stronger negative values of the CUI. CUI values were additively- then log-transformed for generalized linear modeling, and resulting model summary coefficients for predictive variables were back-transformed for interpretation. Solar zenith was extracted for each point and further sorted into the categories of ‘day’ (zenith < 90°), ‘night’ (zenith > 102°) or ‘transitional’ (zenith ≥ 90° or ≤ 102°) [51].

Hauling out behavior in regards to tidal height was investigated via a generalized linear mixed model which also included the explanatory variables of (log) solar zenith, season, upwelling index, and capture location while accounting for capture year. The most parsimonious and explanatory model for hauling out status via the MuMIN ‘dredge’ function in R included all listed variables.

To generally describe space use characteristics of individuals on a two-dimensional, geographic basis in relation to haulout location, we calculated the mean and range in latitude (decimal degrees), and distance from shore (km). Points were categorized as ‘present’ or ‘absent’ within 11 different bays, rivers or estuaries, including the Columbia River, Nehalem Bay, Tillamook Bay, Netarts Bay, Sandlake, Nestucca Bay, Siletz Bay, Depoe Bay, Yaquina Bay, Alsea Bay, and the Siuslaw River.

Static oceanography including bathymetric isopath and three primary categories of bottom substrate (or ‘other’) were extracted for each point. For assessment of bathymetry, locations were categorized to either the 50m, 100m, 150m, 200m, and >200m (off shelf) isobaths using the ArcGIS Spatial Join tool. As harbor seals are generally benthic foragers and use of bottom substrate is likely dependent on foraging strategy and preferred prey, lithography was extracted for each modeled location using the Spatial Join tool in ArcGis for the the Goldfinger et al. OR-WAGeo-HapMaps dataset [52] which had a spatial resolution of 0.5m to 50–100’s of meters. Presence on these substrates and depth ranges were also converted to total percent use by individuals, and use of sandy substrate specifically was examined in a linear mixed effects model against haulout site and season.

### Ethics approvals

This study was carried out in strict compliance with all applicable animal care and use guidelines under the U.S. Animal Welfare Act and was approved as required under the U.S. Marine Mammal Protection Act by the National Marine Fisheries Service (NMFS #16991) and by the Institutional Animal Care and Use Committees of San Jose State University (AUP #1010) and Oregon State University (ACUP #4616).

## Results

Tags transmitted for a mean (±2 S.D.) of 130.25 ± 82.16 days. The minimum period of successful tag transmission was 20 days and the maximum period was 324 days on an alternating duty cycle which excluded the months of January, March, June, August and November for 2014 and 2015 (Table 1). 14% of total raw locations were removed by the SDA filter and burnout period.

**Fig 1:**
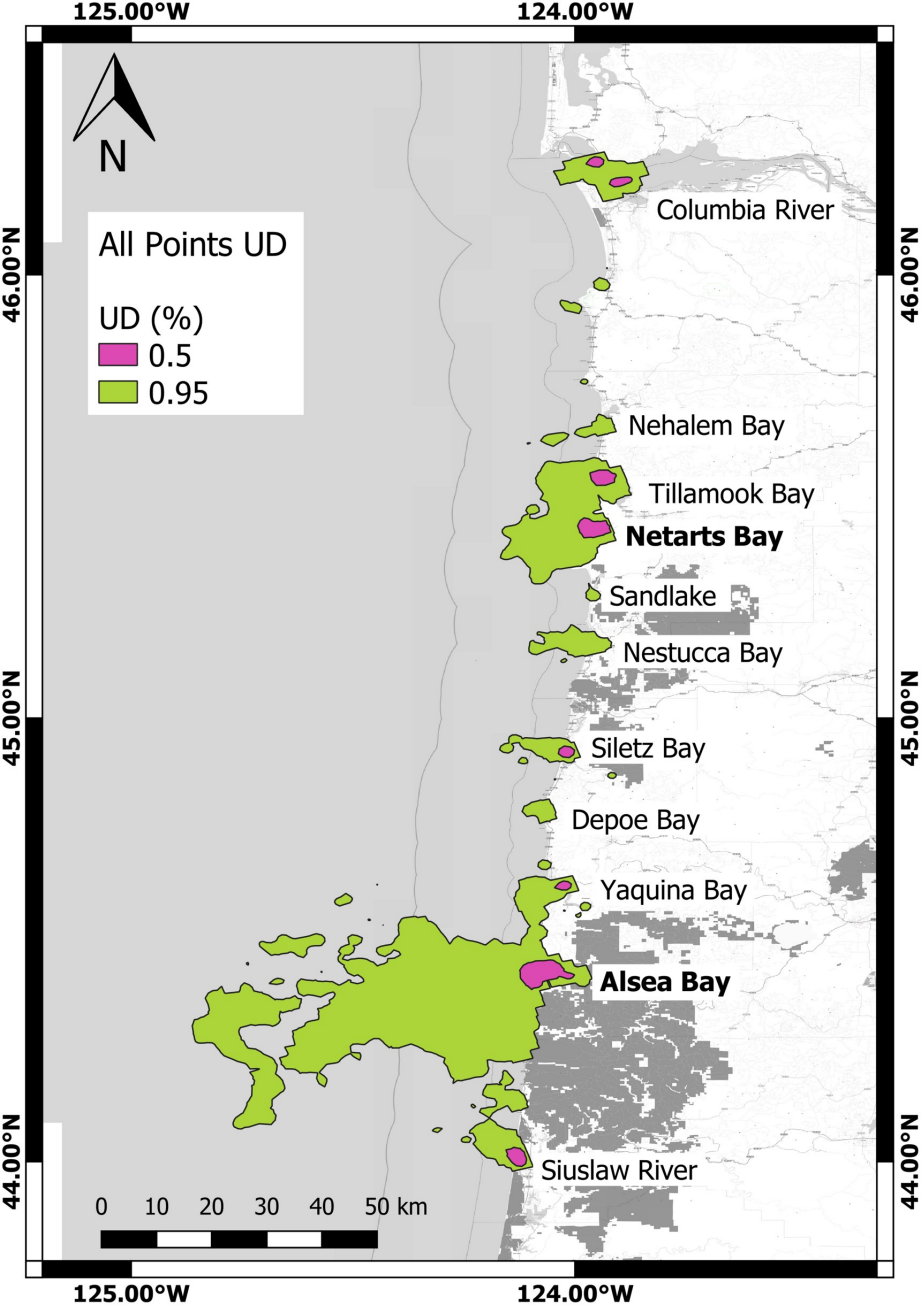
50% and 95% utilization distributions (general probably distribution of encounter for all 24 tagged harbor seals at a 2km x 2km (CVh) resolution. Name of 11 bays and estuaries with seal presence labeled in black letters, with capture sites depicted by bold text. 50% UD labeled in fuchsia, 95% UD labeled in chartreuse (Coordinate system: GCS WGS 1984).

In total, the grouped 95% utilization distribution of all locations included 3,040 square kilometers of the Oregon coast, with a latitudinal range of (43.78 °N, 46.27°N) and a longitudinal range of (-124.96 °W, -123.83°W) (Fig 1). Animals displayed primary use of the continental shelf with less than 0.25% of modeled locations occurring at depths greater than 200 meters. Animals appeared to prefer nearshore areas with 85.34% of modeled locations being within 10km of shore and 44.99% being within one km from shore.

An examination of use of the three most common substrate types (mud, rock, and sand) revealed that sandy substrate had the highest frequency of use (presence). Animals captured in fall demonstrated a significantly higher use of sandy habitats than those tagged in spring (17.94±6.86, *p* = 0.0162). There was no apparent difference (*p >* 0.05) in sandy habitat use between animals captured in Netarts and Alsea Bays.

### Utilization distribution analysis

**Fig 2:**
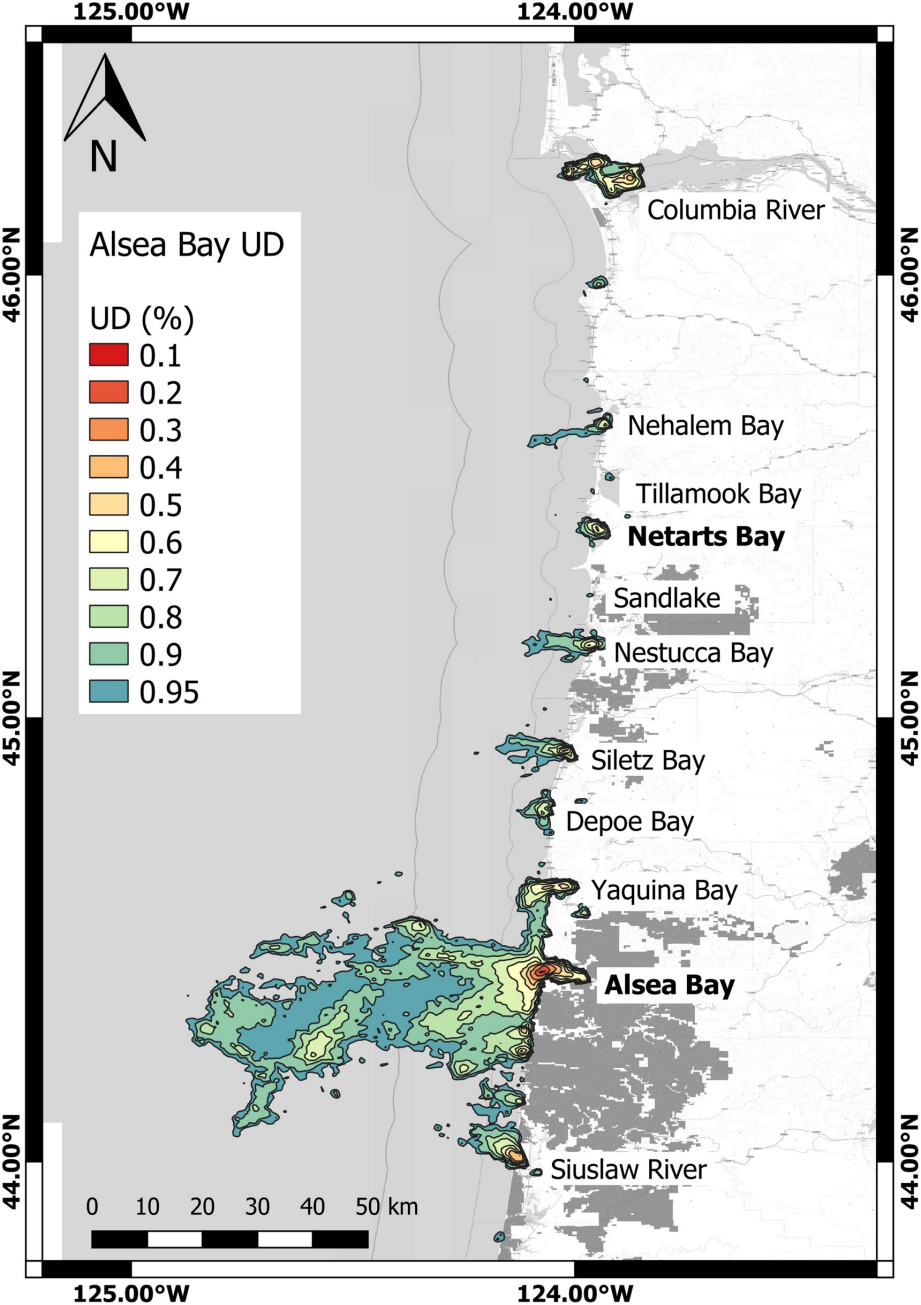
Map of 95% to 10% utilization distribution isopleths (general probability distribution of encounter) at a 2km x 2km (CVh) resolution for seals captured in Alsea Bay (n = 16). Name of 11 bays and estuaries with seal presence labeled in black letters, with capture sites depicted by bold text. Utilization distributions are as shown in legend, with warm colors depicting higher probability of encounter (Coordinate system: GCS WGS 1984).

**Fig 3:**
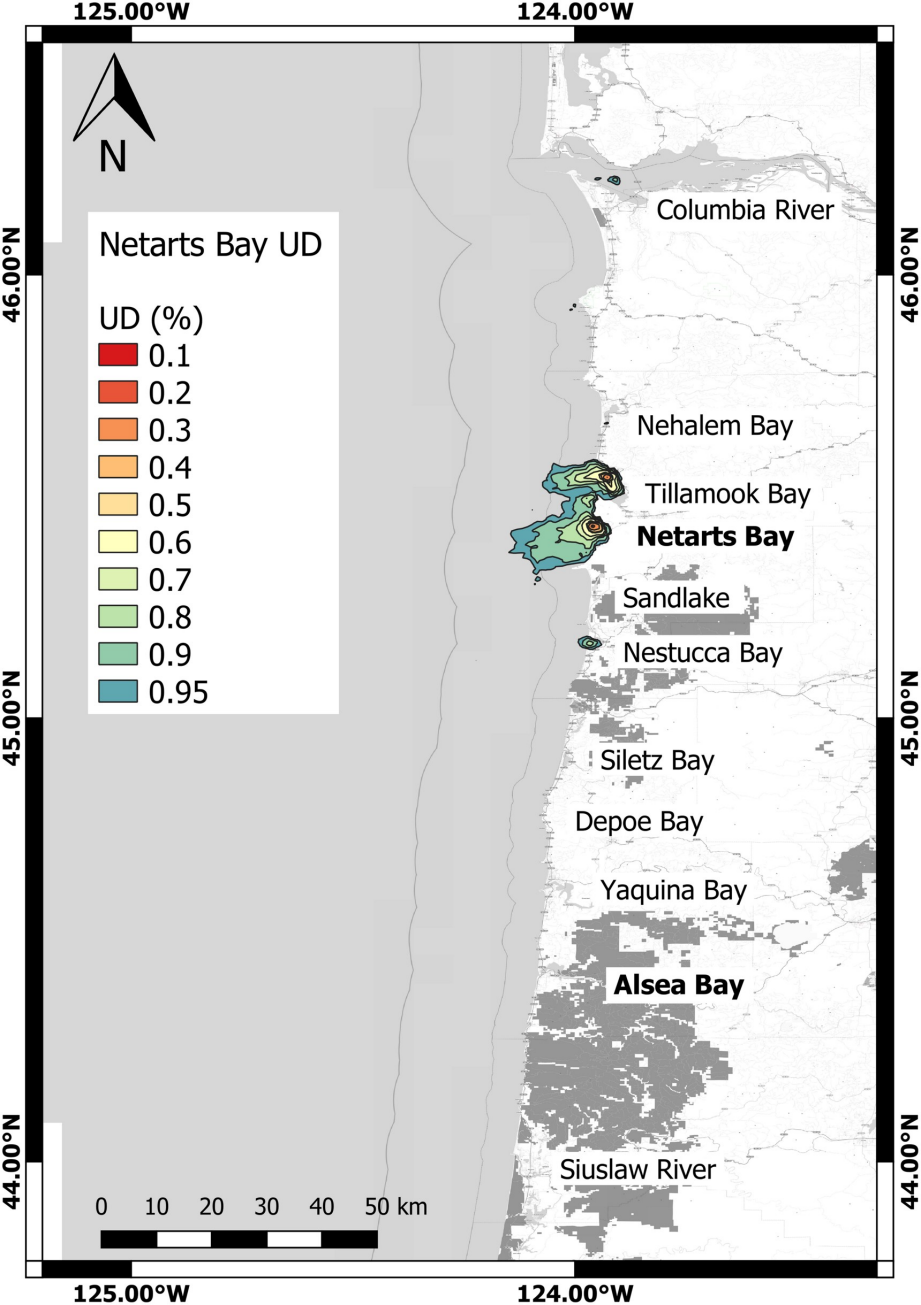
Map of 95% to 10% utilization distribution isopleths (general probability distribution of encounter) at a 2km x 2km (CVh) resolution for seals captured in Netarts Bay (n = 8). Name of 11 bays and estuaries with seal presence labeled in black letters, with capture sites depicted by bold text. Utilization distributions are as shown in legend, with warm colors depicting higher probability of encounter (Coordinate system: GCS WGS 1984).

Kernel density analysis revealed high inter-individual variability in space use (Table 3). The mean individual home range area for all animals (95% UD) was 364.47 ± 382.87 km^2^. Animals tagged in Alsea Bay had a significantly larger average home range than animals tagged in Netarts Bay (Welch’s Two Sample t-test *p* = 0.028, 95% CI = 35.87–577.92, t = 2.35) (Figs 2 and 3). On average, core area represented 29.41 ± 29.23 km^2^, or 11.2% ± 19.3% of home range area for all animals, and 53% of the UD. One seal, #61773, had such a small overall home range (3.20 km^2^) that no core area could be calculated. For all other animals, the relative intensity of use (*I* value) of core area was >1, indicating high intensity of use. Relative intensity of use varied from a value of 2.41 to 59.51, averaging 12.8 ± 14.9. In a linear model of (log)home range area (km^2^) versus deployment days and total SSM locations, (log)home range area was not significantly correlated with these factors (*p* = 0.6029; F-statistic = 0.5183), indicating that the data gathered was sufficient to adequately describe these ranges, and that longer deployments were not associated with larger measured home range.

**Table 3:** Table of home range area, core area, core isopleth, proportion core, isopleth volume, and intensity of use for all study animals (n = 24).

PTT	Home Range Area (km^2^)	Core Area (km^2^)	Core Isopleth	Proportion Core (%)	Isopleth Vol. (%)	Intensity of Use
44611	582.62	30.40	0.50	0.05	0.53	10.15
44613	112.87	1.28	0.40	0.02	0.48	42.57
44614	318.22	70.04	0.60	0.13	0.66	2.98
44615	164.12	1.33	0.40	0.03	0.48	59.51
61694	287.04	17.90	0.50	0.06	0.57	9.14
61695	1647.93	87.01	0.50	0.05	0.54	10.27
61698	363.41	18.95	0.50	0.05	0.58	11.22
61754	1198.02	96.89	0.50	0.08	0.58	7.20
61764	519.53	79.78	0.60	0.08	0.62	4.01
61765	385.58	3.15	0.30	0.02	0.36	43.87
61766	526.24	23.62	0.50	0.04	0.56	12.52
61767	187.33	12.80	0.50	0.07	0.53	7.80
61768	45.23	2.26	0.50	0.05	0.58	11.61
61769	179.10	35.88	0.60	0.11	0.64	3.22
61770	430.09	31.48	0.50	0.07	0.58	7.90
61771	229.97	66.33	0.60	0.19	0.69	2.41
61772	96.75	5.67	0.50	0.06	0.55	9.47
61773	3.20	3.20	0.95	100.00	1.00	1.00
61774	713.83	42.98	0.50	0.06	0.54	8.92
61775	50.83	9.26	0.60	0.07	0.62	3.40
61776	65.87	9.69	0.60	0.10	0.61	4.16
61777	124.15	21.62	0.60	0.10	0.64	3.68
61778	121.03	21.71	0.60	0.14	0.63	3.49
61779	394.26	12.73	0.40	0.06	0.46	14.13

### Intersite differences

Average point distance from shore in kilometers varied significantly for individuals from different tagging locations (Welch’s two-sample t-test *p* = 0.004, 95% CI = 1.91–8.74, t = 3.2995). Animals captured in Alsea Bay on average ventured farther from shore (6.84 ± 6.31km) than Netarts Bay animals (1.51 ± 0.95km). Alsea Bay seals did not cover significantly more distance per 24-hour period on average than seals tagged in Netarts Bay (27.73 ± 11.68km vs. 19.90 ± 14.17km, Welch’s t-test *p* = 0.2015, t = 1.3519). The most commonly used marine reserve was the Cape Perpetua area, approximately 15km south of Alsea Bay, where 1.17% of total SSM-generated locations occurred (S1 Table). Two individuals (#44611 and #61771), both captured in Alsea Bay, spent 28.65% and 18.16% of their time, respectively within this area accounting for most of the total seal presence in the area during this study. In total, 1.33% of all SSM locations occurred within designated reserves with the remaining reserves accounting for only 0.16% of all locations. On average, each animal spent an average of 6.36±10.73 hours per trip in these areas.

### Utilization of bays, rivers and estuaries

In total, 46.70% of all locations were within bays, rivers, or estuaries; 23.44% of these were present within Alsea Bay alone. Netarts Bay accounted for 6.01% of all locations, and presence in Tillamook Bay, directly north of Netarts Bay accounted for 7.5% of all locations. These three bays accounted for 36.95% of all data (S2 Table). Three animals tagged in Alsea Bay were also present in Netarts Bay at some point during the deployment period.

Within bay and riverine environments, seals spent an average of 70.88% of their time in the water (‘wet’), and the remainder of their time ‘dry’. Study animals made a total of 1,618 trips (average per day = 0.62±0.35) within the boundaries of bays, rivers or estuaries during the tag deployment period, spending an average duration of 17.24±80.95 hours within these areas, and spending on average 22.00±24.66 sequential hours in the open ocean.

Seals spent an average of 26.96±15.91 consecutive hours in the water, returning to haul out for an average of 9.43±28.41 hours between trips. Seals were more likely to haul out during lower tides according to a generalized linear mixed model (-0.32±0.01, *p* <0.0001).

## Discussion

The aims of this paper were to quantify home range, use of bays, rivers, estuaries and open ocean habitat, and compare at sea space use of seals from two locations on the Oregon coast. To accomplish this, we analyzed spatial data collected from 24 adult Pacific harbor seals tagged with Wildlife Computers SPOT5 satellite tags in 2014–16. Tags were duty cycled in an effort to provide longer batter life and thus increased tag transmission durations. Duty cycling did come at the potentially significant expense of continuous data. The nature of the on-off monthly duty cycling means that some months during the tag deployment, and the movements undertaken therein, were not accounted for. Some broad seasonal comparisons were made in regards to comparative behavior. However, due to duty cycling and sample size, an emphasis on seasonally-specific comparisons was not entirely appropriate for this analysis and dataset. Future studies with a larger sample size of animals and longer-lasting or renewably-powered tags could more strongly address seasonal patterns in space use, and the use of GPS-based tags would reduce measurement error allowing for finer scale spatial analysis. However, being that this dataset is the first at-sea tracking of multiple harbor seals in Oregon. The data gathered and presented here has value in describing where harbor seals in Oregon go while at sea and how much time they spend in estuaries, rivers, or bays.

The spatial behaviors of harbor seals in this study fell within the general, but widely variable, expectations of spatial behavior for Pacific harbor seals, as well as other subspecies of harbor seal [53–55]. In Alaska, regional harbor seal home ranges, calculated via minimum convex polygons, ranged from as little as 66.2 km^2^ in the icy strait to 7885.1 km^2^ in the Gulf of Alaska [55]. Womble and Gende (2013) noted that while harbor seals in Glacier Bay National Park, Alaska displayed differential movement patterns during and post-breeding season, they also displayed a high degree of inter-individual variablility. While long-distance migration and potential multi-day use of open ocean areas was documented by Womble and Gende, [56], animals in the current study matched other examination of harbor seal behavior and did not appear to display long-distance migratory behavior [57], although one animal, #61779, traveled north to winter in the Columbia River in 2015–16.

In a study of harbor seal movement in the inland waters (Puget Sound and related areas) of Washington state, eight of sixteen harbor seals traveled distances >100km during the study period [58]. These long-distance movements of Washington animals tended to undertaken by males. The female in our study demonstrated home range and core areas that fell well within the distribution of male animals. With a limited sample size, the female was included in this analysis as a valuable asset to understanding species-level basic space use without further comparative analysis between male and female seals. Because of the largely male-skewed sample population, and relatively small sample size, our data cannot be definitely extrapolated to the Oregon harbor seal population as a whole. However, it provides general data that is the first of its kind for this particular region, and is useful for illuminating further questions of interest.

Throughout their range, harbor seals are generally observed in nearshore areas in association with estuarine habitats. This has been demonstrated several times in Oregon via radio telemetry and visual observation [30,32,34]. Our results suggest that seals along the Oregon coast allotted similar amounts of time to hauling out as southern conspecifics in California [9], subject to the same tidal cycles as Oregon. Open ocean trips lasted approximately 22 hours, revealing a pattern of foraging that matched roughly with the full 24.83hr tidal cycle in the eastern North Pacific [59]. Seals in this study were also more likely to have a status of ‘dry’ during lower tidal periods, which generally agrees with what is known about harbor seal hauling out behavior.

We observed strong inter-individual variation in behavior, and significant behavioral differences were measurable between the two tagging locations of Alsea Bay and Netarts Bay, including latitudinal range, distance from shore, home range and core area. The geomorphologies of the continental shelf near Netarts Bay (approximately 20km wide) and of Heceta Bank (approximately 55km wide) directly southwest of Alsea Bay were likely a contributing factor in Alsea Bay seals venturing farther from shore. Individual home range and core area displayed a high level of inter-individual differences that have also been demonstrated in previous studies of harbor seals [60]. Animals in this study were confirmed as nearshore foragers, with the majority of all locations within 10km of shore, and less than 0.25% of modeled locations being beyond the 200m isobath. The most commonly-used estuarine or bay locations used for the animals in the study were Alsea Bay, Netarts Bay, and Tillamook Bay. This strongly points to site fidelity since animals were all captured in the prior two bays, and Tillamook Bay is directly north of Netarts Bay.

Locations from Netarts Bay animals had an overall small average distance from shore. However, the fact that over 85% of locations were within 10km of shore–well within the shelf even for the narrows at Netarts Bay–demonstrates that habitat use was most likely linked to shallower isobaths rather than exploring the full extent of the shelf. This was further confirmed by the fact that almost 45% of locations were within 1km of shore, and over 70% of locations were on, or within 10km of the 50 meter isobaths (the shallowest of four examined isobaths– 50m, 100m, 150m, and ≥200m). Therefore, foraging range may be more closely tied to the extent of shallower isobaths rather than the full range of the continental shelf itself. An alternate hypothesis is that use of habitat type (i.e. substrate) drives space use, rather than bathymetry alone.

Oregon represents a considerable portion of the highly-productive Northern California Current System. The California Current System as a whole is one of four major Eastern Boundary Current Systems, all of which are particularly rich regions of biodiversity and fisheries production [61]. Model-based studies of this system have traditionally focused on lower and mid-trophic levels [62–64], or non-marine mammal predators [65]. The study goes beyond these to examine habitat use by a common air breathing marine predator.

Various dynamic changes in northern California Current System have taken place since the previous monitoring, and lower population levels, of harbor seal populations. These include shifts in climatological patterns and nutrient transport [66] and deoxygenation and hypoxic events [43,67,68] which will likely continue to accelerate in the future, with the potential for large-scale changes in ecosystem structure and functioning. Drastic population increases since enactment of the MMPA also merits re-examination and understanding of the ecology of these animals, as existing studies on harbor seal ecology and movement took place before these population increases. Additionally management of harbor seals with the state of Oregon is largely based on the assumption that all animals fall under the same ecological and genetic stock [1,69]. However, these previous assessments often examined animals from individual sites and did not compare animals across haulouts. Therefore, working to understand the behavioral diversity of animals along the coast is key to understanding the full ecology of this species.

Since 2012, five marine reserves have been established in this system along the Oregon coast, in conjunction with eight marine protected areas, one seabird protection area, and nine associated comparison areas. Only two of 24 animals in this sample were present within these marine reserves more than 10% of the time. Our data did not provide any evidence of intensive use of marine protected areas or reserves. The majority of presence within reserve areas was within Cape Perpetua Marine Reserve and its associated northern Marine Protection Area, potentially due to both geographic proximity to the haulout and the highly productive nature of Heceta Bank on which it is located. However, findings regarding use of bathymetry and habitat also suggest that the particular habitat makeup of a conservation area may strongly affect how it is used by marine mammals. This data, though limited, does provide some evidence of use of Oregon’s marine reserves by particular individual seals and is the first documented use of these management areas by pinnipeds at sea. Resultant marine mammal presence, and subsequent predation on fish species within marine management areas could be a topic of further in-depth examination.

Overall, this study revealed previously unexamined habitat use for a single species of generalist marine mammal in a region of the northern California Current System. The data presented here lends itself as a baseline data of harbor seal space use in relation to coastal geography and management areas that can be used to generate further hypotheses and draw future seasonal, inter-annual, and inter-regional comparisons.

## Supporting information

S1 Table Percent of total SSM locations within 7 MPAs or seabird PAs in Oregon. In total, 1.55% of data points (n = 886) were classified as present within one of these area.
(DOCX)

S2 Table Percent of total state space modeled locations present within 11 coastal bodies of water in Oregon. In total, 47.60% of data points (n = 27,235) were classified as present within one of these areas. Gray represents animals tagged in Alsea Bay, white represents animals tagged in Netarts Bay.
(DOCX)
