# Supplementary material for: Space use of Pacific harbor seals (Phoca vitulina richardii) from two haulout locations along the Oregon coast
Source: PLoS One. 2019 Jul 31;14(7):e0219484. doi: 10.1371/journal.pone.0219484 (PMC6668786; doi:10.1371/journal.pone.0219484)
Supplement: S1 Table — In total, 1.55% of data points (n = 886) were classified as present within one of these area. (DOCX) [file pone.0219484.s001.docx]

**Supp Table 1.**

| ***Ptt*** | ***Total # Locations*** | ***Cape Perpetua North MPA*** | ***Cape Perpetua Seabird Protection Area*** | ***Cape Perpetua SE MPA*** | ***Cape Falcon West MPA*** | ***Cascade Head North MPA*** | ***Cascade Head South MPA*** | ***Cascade Head West MPA*** |
| --- | --- | --- | --- | --- | --- | --- | --- | --- |
| ***44611*** | 1330 | 1.13 (n = 15) |  |  |  |  |  |  |
| ***44613*** | 3425 | 0.32 (n = 11) |  |  |  |  |  |  |
| ***44614*** | 2786 | 0.04 (n = 1) |  |  |  |  |  |  |
| ***44615*** | 1039 | 3.46 (n = 36) |  |  |  |  |  |  |
| ***61694*** | 1927 |  | 6.80 (n = 131) | 4.41 (n = 85) |  |  |  |  |
| ***61695*** | 2848 |  |  |  |  | 0.49 (n = 14) | 1.09 (n = 31) | 0.07 (n = 2) |
| ***61698*** | 1339 | 0.22 (n = 3) | 0.22 (n = 3) |  |  |  |  |  |
| ***61754*** | 2679 | 0.15 (n = 4) |  |  |  |  |  |  |
| ***61764*** | 2661 | 15.30 (n = 407) |  |  |  |  |  |  |
| ***61765*** | 3288 | 0.06 (n = 2) |  |  |  |  |  |  |
| ***61766*** | 4114 | 0.02 (n = 1) |  |  |  |  |  |  |
| ***61767*** | 3759 | 0.19 (n = 7) | 0.56 (n = 21) | 0.27 (n = 10) |  |  |  |  |
| ***61768*** | 1956 |  |  |  |  |  |  |  |
| ***61769*** | 2332 |  |  |  |  |  |  |  |
| ***61770*** | 2292 | 0.57 (n = 13) |  |  |  |  |  |  |
| ***61771*** | 991 | 7.47 (n = 74) | 1.11 (n = 11) |  |  |  |  |  |
| ***61772*** | 1587 |  |  |  |  |  |  |  |
| ***61773*** | 1543 |  |  |  |  |  |  |  |
| ***61774*** | 1967 |  |  |  | 0.15 (n = 3) |  |  |  |
| ***61775*** | 4208 |  |  |  |  |  |  |  |
| ***61776*** | 1227 |  |  |  |  |  |  |  |
| ***61777*** | 474 |  |  |  |  |  |  |  |
| ***61778*** | 2133 |  |  |  |  |  |  |  |
| ***61779*** | 531 |  |  |  |  |  | 0.19 (n = 1) |  |
| ***SUM*** | **57220** | **2.11**  **(n = 574)** | **0.29**  **(n = 166)** | **0.17**  **(n = 95)** | **0.01**  **(n = 3)** | **0.02**  **(n = 14)** | **0.06**  **(n = 32)** | **<0.01**  **(n = 2)** |
