# Supplementary material for: Space use of Pacific harbor seals (Phoca vitulina richardii) from two haulout locations along the Oregon coast
Source: PLoS One. 2019 Jul 31;14(7):e0219484. doi: 10.1371/journal.pone.0219484 (PMC6668786; doi:10.1371/journal.pone.0219484)
Supplement: S2 Table — In total, 47.60% of data points (n = 27,235) were classified as present within one of these areas. Gray represents animals tagged in Alsea Bay, white represents animals tagged in Netarts Bay. (DOCX) [file pone.0219484.s002.docx]

**Supp Table 2.**

| ***Ptt*** | ***Total # Locations*** | ***Alsea Bay*** |  | ***Columbia River*** | ***Depoe Bay*** | ***Nehalem Bay*** | ***Nestucca Bay*** | ***Netarts Bay*** | ***Sand Lake*** |  | ***Siletz Bay*** | ***Siuslaw River*** | ***Tillamook Bay*** | ***Yaquina Bay*** | ***Total*** |
| --- | --- | --- | --- | --- | --- | --- | --- | --- | --- | --- | --- | --- | --- | --- | --- |
| ***44611*** | 1330 | 15.34 (n=204) |  |  |  |  |  |  |  |  |  |  |  |  | 15.34 (n=204) |
| ***44613*** | 3425 | 51.88 (n=1777) |  |  |  |  |  |  |  |  |  |  |  |  | 51.88 (n=1777) |
| ***44614*** | 2786 | 18.59 (n=518) |  |  |  |  |  |  |  |  |  |  |  | 0.97 (n=27) | 19.56  (n=545) |
| ***44615*** | 1039 | 53.80 (n=559) |  |  |  |  |  |  |  |  |  |  |  |  | 53.80 (n=559) |
| ***61694*** | 1927 | 38.30 (n=738) |  |  |  |  |  |  |  |  |  | 3.84 (n=74) |  | 0.36 (n=7) | 42.50  (n=819) |
| ***61695*** | 2848 | 4.60 (n=131) |  | 10.04  (n=286) | 0.04 (n=1) | 3.69 (n=105) | 13.65 (n=389) | 1.58 (n=45) | 0.11 (n=3) |  | 13.66 (n=389) |  | 0.46 (n=13) |  | 47.82  (n=1362) |
| ***61698*** | 1339 | 42.12 (n=564) |  |  |  |  |  | 7.99 (n=107) |  |  |  |  |  |  | 50.11  (n=671) |
| ***61754*** | 2679 | 22.84 (n=612) |  |  | 1.38 (n=37) |  |  |  |  |  |  |  |  | 0.26 (n=7) | 24.49  (n=656) |
| ***61764*** | 2661 | 29.20 (n=777) |  |  |  |  |  |  |  |  |  |  |  | 0.08 (n=2) | 29.27  (n=779) |
| ***61765*** | 3288 | 79.68 (n=2620) |  |  |  |  |  |  |  |  |  | 0.79 (n=26) |  |  | 80.47  (n=2646) |
| ***61766*** | 4114 | 40.62 (n=1671) |  |  |  |  |  |  |  |  |  |  |  | 0.32 (n=13) | 40.93  (n=1684) |
| ***61767*** | 3759 | 16.73 (n=629) |  |  |  |  |  |  |  |  |  | 27.96 (n=1051) |  |  | 44.69  (n=1680) |
| ***61768*** | 1956 |  |  |  |  |  |  |  |  |  |  |  | 77.97 (n=1525) |  | 77.97 (n=1525) |
| ***61769*** | 2332 |  |  |  |  |  |  |  |  |  |  |  | 0.99 (n=23) | 0.04 (n=1) | 1.03  (n=24) |
| ***61770*** | 2292 | 41.67 (n=955) |  |  |  |  |  |  |  |  |  |  |  |  | 41.67 (n=955) |
| ***61771*** | 991 | 10.09 (n=100) |  |  |  |  |  |  |  |  |  |  |  |  | 10.09 (n=100) |
| ***61772*** | 1587 |  |  |  |  |  |  | 43.23 (n=686) |  |  |  |  |  |  | 43.23 (n=686) |
| ***61773*** | 1543 |  |  |  |  |  |  | 73.36 (n=1132) |  |  |  |  |  |  | 73.36 (n=1132) |
| ***61774*** | 1967 |  |  | 5.59  (n=110) |  | 0.56 (n=11) |  | 23.95 (n=471) | 0.31 (n=6) |  |  |  | 0.86 (n=17) |  | 31.27  (n=615) |
| ***61775*** | 4208 |  |  |  |  |  |  | 1.45 (n=61) |  |  |  |  | 60.27 (n=2536) |  | 61.72  (n=2597) |
| ***61776*** | 1227 |  |  |  |  |  |  | 33.90 (n=416) |  |  |  |  | 11.82 (n=145) |  | 45.72  (n=561) |
| ***61777*** | 474 |  |  |  |  |  |  | 44.30 (n=210) |  |  |  |  | 3.16 (n=15) |  | 47.47  (n=225) |
| ***61778*** | 2133 | 17.25 (n=368) |  |  |  |  |  |  |  |  |  |  |  | 36.19  (n=772) | 53.45  (n=1140) |
| ***61779*** | 5315 | 22.37 (n=1189) |  | 52.25  (n=2777) |  |  |  | 5.85 (n=311) |  |  |  |  | 0.30 (n=16) | 0.02  (n = 1) | 80.77  (n=4293) |
| ***SUM*** | **57220** | **23.44 (n=13412)** |  | **5.55 (n=3173)** | **0.07 (n=38)** | **0.20 (n=116)** | **0.68 (n=389)** | **6.01 (n=3439)** | **0.02 (n=9)** |  | **0.67 (n=389)** | **2.01 (n=1151)** | **7.50 (n=4290)** | **1.45 (n=829)** |  |
